# Cytokeratin 18, Alanine Aminotransferase, Platelets and Triglycerides Predict the Presence of Nonalcoholic Steatohepatitis

**DOI:** 10.1371/journal.pone.0082092

**Published:** 2013-12-04

**Authors:** Wei Cao, Caiyan Zhao, Chuan Shen, Yadong Wang

**Affiliations:** Department of Infectious Diseases, The Third Hospital of Hebei Medical University, Shijiazhuang, China; Inserm, U1052, UMR 5286, France

## Abstract

**Background:**

Nonalcoholic fatty liver disease (NAFLD) is one of the critical public health problems in China. The full spectrum of the disease ranges from simple steatosis and nonalcoholic steatohepatitis (NASH) to cirrhosis and hepatocellular carcinoma(HCC). The infiltration of inflammatory cells characterizes NASH. This characteristic contributes to the progression of hepatitis, fibrosis, cirrhosis, and HCC. Therefore, distinguishing NASH from NAFLD is crucial.

**Objective and Methods:**

Ninety-five patients with NAFLD, 44 with NASH, and 51 with non-NASH were included in the study to develop a new scoring system for differentiating NASH from NAFLD. Data on clinical and biological characteristics, as well as blood information, were obtained. Cytokeratin-18 (CK-18) fragments levels were measured using an enzyme-linked immunosorbant assay.

**Results:**

Several indexes show significant differences between the two groups, which include body mass index (BMI), waist-on-hip ratio (WHR), aspartate aminotransferase (AST), alanine aminotransferase (ALT), alkaline phosphatase (ALP), γ-glutamyl transpeptidase (γ-GT), platelets, uric acid (UA), hs-C-reactive protein (hs-CRP), triglycerides (TG), albumin (ALB), and CK-18 fragments (all *P* < 0.05). The CK-18 fragment levels showed a significant positive correlation with steatosis severity, ballooning, lobular inflammation, and fibrosis stage (all *P* < 0.05). Therefore, a new model that combines ALT, platelets, CK-18 fragments, and TG was established by logistic regression among NAFLD patients. The AUROC curve in predicting NASH was 0.920 (95% CI: 0.866 - 0.974, cutoff value = 0.361, sensitivity = 89%, specificity = 86%, positive predictive value = 89%, negative predictive value = 89%).

**Conclusion:**

The novel scoring system may be considered as a useful model in predicting the presence of NASH in NAFLD patients.

## Introduction

Nonalcoholic fatty liver disease (NAFLD) is a clinic-pathological syndrome with a full spectrum that ranges from simple steatosis and nonalcoholic steatohepatitis (NASH) to cirrhosis and hepatocellular carcinoma [1,2]. In China, NAFLD is a crucial public health problem that affects approximately 15% of the general population [3,4]. Strong etiologic and chronologic associations are observed with metabolic syndrome manifestations, including hypertension, diabetes mellitus or impaired fasting glycemia, hyperlipidemia, and central obesity [5]. Majority of NAFLD cases are simple steatosis, which typically follows a benign, non-progressive clinical course [6]. NASH is a more aggressive form of NAFLD, which is characterized by steatosis, hepatocellular injury, lobular inflammation, and the pattern of fibrosis [7,8], and is a potential cause of cirrhosis and end-stage liver disease [9]. About 20% of NASH patients progress to cirrhosis, whereas one-third of early-stage NASH will progress to stage 3 or 4 (cirrhosis) in 5 to 10 years [10]. Approximately 30% to 40% of NASH patients with cirrhosis undergo receive a liver transplant or die of liver-related complications [11,12]. Thus, distinguishing patients with NASH from simple steatosis is important to guide therapies and to determine potential risks on the progression of liver disease.

Most NAFLD are diagnosed by combining several imaging methodologies including ultrasonography, computer tomography scan, and magnetic resonance imaging. However, hepatic fibrosis is inaccurately detected accurately by imageological methods alone. The degree of liver fibrosis is recently measured by transient elastography (FibroScan), which is a simple, accurate, quick, and novel noninvasive method that has been validated in patients with NAFLD [13]. However, this method is not applicable in some obese patients because the subcutaneous fat attenuates the transmission of shear waves into the liver [14,15]. The method has excellent negative predictive value in excluding advanced fibrosis and cirrhosis among NAFLD patients, whereas, the positive predictive value in diagnosing advanced disease remains modest [16]. Thus, these noninvasive techniques cannot completely replace biopsy, and the identification of simple steatosis from NASH by medical imaging techniques remains difficult. Liver biopsy, which is considered as the gold standard in assessing hepatic pathology in NAFLD, poses several limitations because this method is invasive, local, and expensive. Thus, the development of noninvasive markers to differentiate NASH from simple steatosis is necessary in clinical settings. The development would free patients from liver biopsies.

Various factors influence the pathogenesis of NAFLD, such as insulin resistance, abnormal lipid, glucose metabolism, oxidative stress, iron overload, hepatic inflammation, apoptosis, and et al [17-20]. Apoptosis is an active ATP-dependent process that contributes to the maintenance of tissue homeostasis under normal physiological conditions [21]. Cytokeratin-18 (CK-18) is an intracellular protein mainly produced by the necrosis and apoptosis cells of epithelial origin including hepatocytes, and is a useful serum biomarker that reflects cell death [9]. The M30 antibody generated in the execution phase of apoptosis is commonly used to identify an apoptosis-specific neoepitope at the CK-18 aspartic acid residue 396 [22]. The increased serum levels of CK-18 fragments in some clinical cohorts of patients with obesity or insulin resistance were associated with hepatocytic injury, inflammation, and fibrosis. Thus CK-18 was proposed as a potential biomarker to predict the liver histological manifestation of NASH [23-27]. 

In China, NAFLD is mainly diagnosed through physical examination, especially ultrasonography. Biomarkers are often included in regular examinations to establish a noninvasive scoring system for the differentiation between NAFLD and NASH. The selected markers were associated with the physiopathological mechanisms of NAFLD. Therefore, this study investigate the correlations among serum CK-18 fragments, clinical data (including markers closely related to NAFLD and part of the regular examination), and degrees of liver histologies in NAFLD patients. We also attempt to develop a simple noninvasive scoring system to differentiate NASH from NAFLD by combining several serum biomarkers. 

## Materials and Methods

### Ethics Statement

The study was approved by the Ethics Committee of the Third Hospital of Hebei Medical University. The individuals involved signed an informed consent. 

### Study Population and Design

We selected patients from April 10, 2009 to December 1, 2011 in the Third Hospital of Hebei Medical University. The selected patients were diagnosed with NAFLD through clinical manifestations or liver biopsy based on the guideline for diagnosis and treatment of NAFLD by The Chinese National Workshop on Fatty Liver and Alcoholic Liver Disease for the Chinese Liver Disease Association [28]. The sample size (90% power and 95% confidence) was calculated according to every marker in the preliminary experiment data and relevant literature. A total of 95 patients were enrolled in this study. The exclusion criteria are listed as follows: (a) alcoholic fatty liver disease (alcohol consumption ≥ 40 g/d for male or ≥ 20 g/d for female during past five years) or excessive alcohol consumption (≥ 140 g/wk for male or ≥ 70 g/wk for female); (b) viral hepatitis; (c) autoimmune liver diseases; (d) drug- or toxin-induced liver steatosis (with no drugs or toxins which can induce hepatotoxicity); (e) genetic or metabolic liver diseases, such as Wilson’s disease, hemochromatosis, and alfa-1 antitrypsin deficiency; (f) biliary obstruction (by ultrasonography). The patients were divided into two groups, namely, non-NASH and NASH (by liver histology).

### Clinical and Laboratory Assessments

Data on age, gender, and smoking habits (0 = never; 1 = ex; 2 = current;) were obtained via interview. Height, weight, and waist and hip measurements were obtained while standing with light clothing. Height and weight were measured without shoes to the nearest 0.1 cm and 0.1 kg, respectively. 

Blood was obtained from each patient on the same day of liver biopsy. The blood samples were stored at -80 °C. The laboratory evaluation in all patients included: aspartate aminotransferase (AST), alanine aminotransferase (ALT), albumin (ALB), bilirubin, alkaline phosphatase (ALP), γ-glutamyl transpeptidase (γ-GT), platelets, white blood cell (WBC), hemoglobin, creatinine, INR, Uric acid (UA), hs-C-reactive protein (hs-CRP), ferritin, fasting glucose (FG), triglycerides (TG), and total cholesterol (TC). 

Diabetes mellitus (DM) was diagnosed based on medical history and/or FG of 126mg/dL or greater, and hypertension based on blood pressure at ≥ 140/90 mmHg at least twice a day or the use of anti-hypertension drugs. The presence of dyslipidemia was assessed by medical history and/or fasting TC > 200 mg/dL and/or fasting TG > 150 mg/dL. Body mass index (BMI) was calculated as weight (kilograms) divided by height (meters) squared. Waist-on-hip ratio (WHR) = waist/hip.

### Liver Histology

Liver biopsy samples under Haematoxylin-eosin and masson trichrome staining were examined by two pathologists, who were unaware of the clinical and biochemical conditions of each patient. The pathologists provided the pathologic diagnosis based on the criteria reported by Brunt et al [29]. The histological features of the liver were scored according to a histological scoring system for NAFLD [30] [steatosis (0 = <5%; 1 = 5-33%; 2 = 34% to 66%; 3 = >66%;), lobular inflammation (0 = no foci; 1 = <2 foci per 200 x field; 2 = 2 to 4 foci per 200 x field; 3 = >4 foci per 200 x field;), ballooning (0 = none; 1 = rare or few; 2 = many;), fibrosis score (0 = no fibrosis; 1 = mild/moderate perisinusoidal or portal fibrosis; 2 = both perisinusoidal and portal fibrosis; 3 = bridging fibrosis; 4 = cirrhosis;)]. Controversial results were resolved by conferring with each other. A total of 95 patients were divided into two histological groups, namely, non-NASH and NASH group. The former included simple steatosis (steatosis in the absence of inflammation and ballooning hepatocyte degeneration) and borderline/possible NASH (steatosis with minimal, rare inflammation and hepatocyte ballooning), whereas the latter included NASH (steatosis with inflammation and hepatocyte ballooning, often with fibrosis) [31]. 

### Measurement of caspase-generated CK-18 fragments

 CK-18-Asp396 (M30 antigen) is a caspase-degraded product of CK-18 produced by apoptotic cells. The level of serum CK-18-Asp396 was detected by ELISA kit (PEVIVA, Alexis, Grünwald, Germany). Absorbance was determined by microplate reader (TECAN Infinite 200, Austria). 

### Statistical Analysis

Descriptive statistics were computed for all variables. The results were presented as means ± standard deviation for normal continuous data. The differences between non-NASH and NASH were assessed using two-sample t-test. Non-normal continuous data were expressed as medians (25^th^ - 75^th^ percentiles). Comparisons were conducted using the Mann-Whitney U test. The frequencies and percentages were estimated, and Chi-square tests or Fischer’s exact tests were used for categorical variables. Kappa statistic was applied to evaluate agreement between pathologists. Spearman’s correlation coefficient was used to estimate the associations between serum CK-18 fragment levels and several relevant factors. Multivariate analyses were performed using binary logistic regression with the estimation of odds ratios (OR) and 95% confidence intervals (95% CI). Logistic regression was used to identify the independent predictors of NASH. Inclusion of variables was assessed using a stepwise selection method. The receiver operating characteristic (ROC) curve and the area under ROC (AUROC) curve analysis were used to assess the utility of parameters in the diagnosis of NASH. A P value of 0.05 was considered statistically significant. SPSS version 13.0 (SPSS Inc., Chicago, IL, USA) was used to perform all analyses.

## Results

### Characteristics of the study population

Table 1 describes the main clinical and laboratory characteristics of the patients. Compared with the non-NASH group, the levels of BMI, WHR, AST, ALT, ALP, γ-GT, platelets, UA, hs-CRP, and TG were significantly higher in the NASH group (all *P* < 0.05), whereas ALB was lower (*P* < 0.05). Numerous indexes showed no significant differences between the two groups. These indexes include the age of patient, gender, smoking habits, systolic blood pressure (SBP), diastolic blood pressure (DBP), serum bilirubin, WBC, hemoglobin, creatinine, INR, Ferritin, FG, TC, and the medical history of DM, hypertension, and dyslipidemia.

**Table 1:** Clinical and serological characteristics of the patient population.

**Factor**	**All Subjects(n=95)**	**non-NASH(n=51)**	**NASH(n=44)**	***P* value**
Age(years)	48 (40, 60)	54 (41, 63)	45.5 (40, 57)	0.059
Gender(M/F)	22/73	12/39	10/34	0.884
Smoking habits				0.532
0	40 (42.1)	19 (37.3)	21 (47.7)	
1	33 (34.7)	21 (41.2)	12 (27.3)	
2	22 (23.2)	11 (21.5)	11 (25.0)	
BMI(kg/m^2^)	28.5±2.8	27.7±2.5	29.6±2.9	0.001
WHR	0.9 (0.9, 1.0)	0.9 (0.9, 0.9)	0.9 (0.9, 1.0)	<0.001
SBP(mmHg)	130.0 (122.0, 140.0)	138.0 (122.0, 148.0)	130.0 (120.5, 140.0)	0.367
DBP(mmHg)	86.0 (80.0, 90.0)	86.0 (80.0, 90.0)	85.0 (80.0, 93.0)	0.737
AST(U/L)	48.0 (44.0, 54.0)	44.0 (43.0, 48.0)	53.0 (46.5, 59.8)	<0.001
ALT(U/L)	57.0 (48.0, 71.0)	50.0 (45.0, 59.0)	69.5 (58.0, 85.0)	<0.001
ALB(g/dL)	4.7 (4.5, 4.9)	4.8 (4.6, 4.9)	4.7 (4.3, 4.9)	0.035
Bilirubin (mg/dL)	12.1 (9.9, 15.5)	11.7 (9.9, 14.0)	12.6 (10.3, 16.1)	0.243
ALP(IU/L)	96.1±20.2	91.4±20.0	101.6±19.2	0.014
γ-GT(IU/L)	35.0 (27.0, 55.0)	29.0 (25.0, 44.0)	42.0 (33.0, 73.8)	0.001
Platelets(x 10^9^/L)	230.5±51.1	220.9±48.3	241.6±52.6	0.048
WBC(x 10^9^/L)	6.6±1.6	6.4±1.5	6.7±1.7	0.365
Hemoglobin (g/dL)	150.8±12.3	149.7±10.7	152.1±13.9	0.349
Creatinine(mg/dL)	74.1±14.6	75.6±15.6	72.5±13.4	0.306	
INR	1.0±0.1	1.0±0.1	1.0±0.1	0.593	
UA(mg/dL)	5.2 (4.2, 6.3)	5.0 (4.1, 5.9)	5.6 (4.3, 6.9)	0.035	
hs-CRP(mg/L)	2.0 (1.5, 2.7)	1.7 (1.3, 2.6)	2.1 (1.6, 3.0)	0.042	
Ferritin (ng/mL)	177.1±71.9	165.0±63.8	191.1±78.8	0.078	
FG(mg/dL)	99.5 (92.3, 116.6)	100.9 (93.3, 118.6)	98.3 (91.7, 111.5)	0.483	
TG(mg/dL)	204.5 (143.5, 309.1)	160.3 (112.5, 253.3)	257.3 (188.9, 418.9)	<0.001	
TC(mg/dL)	207.3 (185.6, 250.2)	206.8 (189.5, 219.7)	210.3 (177.8, 309.2)	0.201	
DM	23(24.2)	13(25.5)	10(22.7)	0.754	
Hypertension	46(48.4)	28(54.9)	18(40.9)	0.174	
Dyslipidemia	82(86.3)	42(82.4)	40(90.9)	0. 226	

The statistics presented are means ± SD or medians (25^th^-75^th^ percentiles) or N (%).

Abbreviations: N, number of subjects; M, male; F, female; BMI, body mass index; WHR, waist-on-hip ratio; SBP, systolic blood pressure; DBP, diastolic blood pressure; AST, aspartate aminotransferase; ALT, alanine aminotransferase; ALB, albumin; ALP, alkaline phosphatase; γ-GT, γ-glutamyl transpeptidase; WBC, white blood cell; INR, international normalized ratio; UA, uric acid; hs-CRP, hs-C-reactive protein; FG, fasting glucose; TG, triglycerides; TC, total cholesterol; DM, diabetes mellitus;

*P* values correspond to the comparison of the two groups. Two-sample t-test or Wilcoxon rank sum tests for continuous factors and chi-square tests or Fischer’s exact tests were utilized for the categorical factors used.

Table 2 summarizes the histological characteristics, which include specimen length, number of portal tracts, steatosis, inflammation, hepatocyte ballooning, and fibrosis. Participants included 51 patients (54%) without NASH and 44 (46%) with NASH. The Kappa value among the participating pathologists was 0.874.

**Table 2:** Histological characteristics of all patients.

**Factor**	**All Subjects (n=95)**	**non-NASH (n=51)**	**NASH (n=44)**	***P* value**
Specimen length	20 (20, 22)	20 (20, 22)	22 (20, 22)	0.069
Number of portal tracts	13 (11, 15)	12 (11, 14)	13 (10, 16)	0.213
Steatosis severity				<0.001
0	0 (0)	0 (0)	0 (0)	
1	49 (51.6)	38 (74.5)	11 (25)	
2	23 (24.2)	13 (25.5)	10 (22.7)	
3	23 (24.2)	0 (0)	23 (52.3)	
Ballooning				<0.001
0	2 (2.1)	2 (3.9)	0 (0)	
1	77 (81.1)	46 (90.2)	31 (70.5)	
2	16 (16.8)	3 (5.9)	13 (29.5)	
Lobular inflammation				<0.001
0	1 (1.1)	1 (2.0)	0 (0)	
1	43 (45.3)	35 (68.6)	8 (18.2)	
2	33 (34.7)	15 (29.4)	18 (40.9)	
3	18 (18.9)	0 (0)	18 (40.9)	
Fibrosis stage				0.008
0	10 (10.5)	8 (15.7)	2 (4.5)	
1	31 (32.6)	14 (27.4)	17 (38.6)	
2	23 (24.2)	13 (25.5)	10 (22.8)	
3	31 (32.6)	16 (31.4)	15 (34.1)	
4	0 (0)	0 (0)	0 (0)	

Statistics presented are medians (25^th^-75^th^ percentiles) or N (%).

Abbreviations: N, number of subjects;

*P* value corresponds to Mann-Whitney U tests.

### The serum level of CK-18 fragments markedly increased in patients with NASH

**Figure 1:**
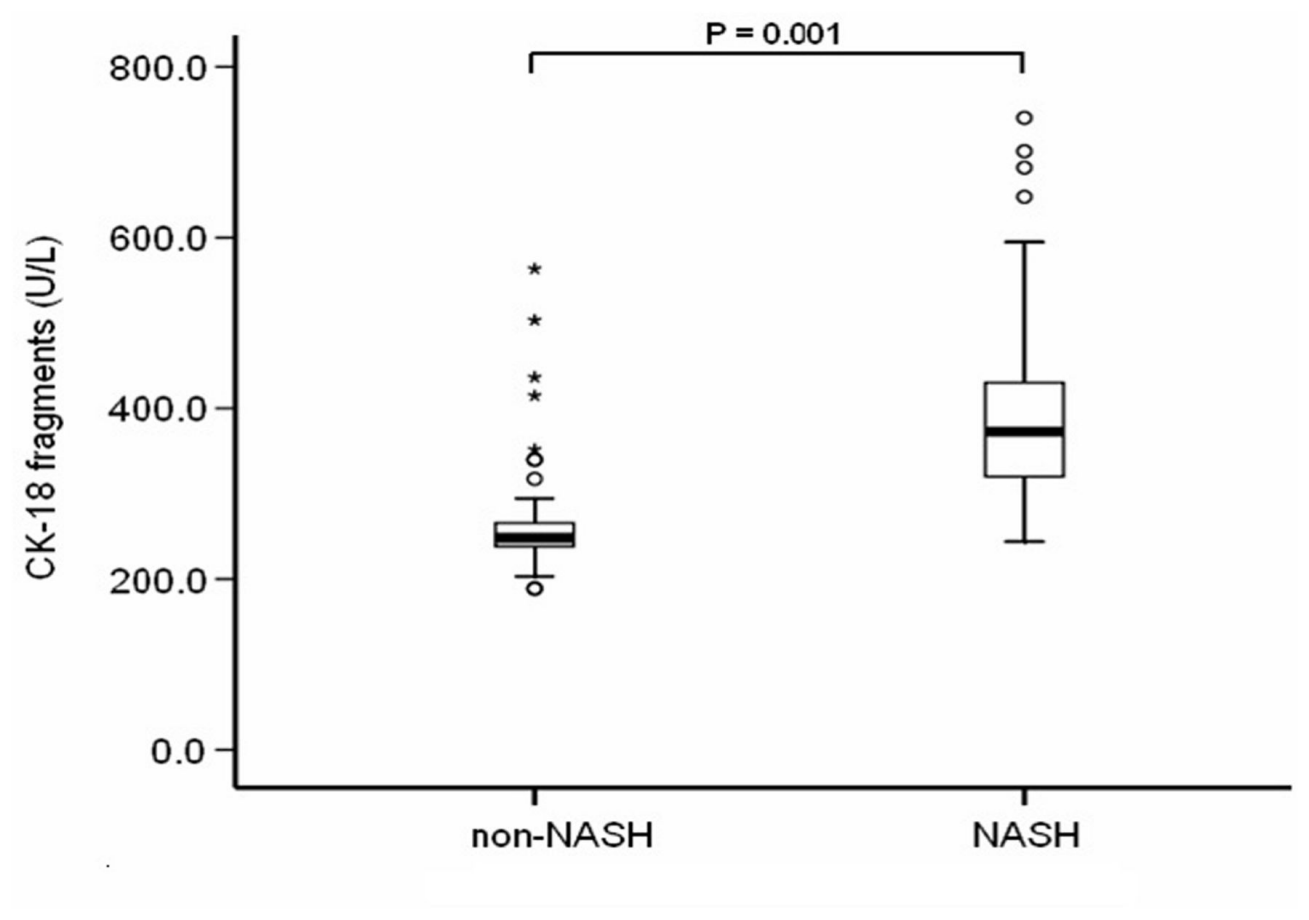
CK-18 fragments are significantly higher in the serum with NASH compared to patients with non-NASH. The vertical axis represents serum CK-18 levels in U/L, whereas the horizontal axis refers to patient groups. The box and bars represent the interquartile range (the 25^th^ and 75^th^ percentiles) from the median (the horizontal line) and the 95% confidence interval, respectively. CK-18 fragments levels significantly increased in patients with NASH compared to non-NASH patients (median (Q25, Q75): 372.9 U/L (319.6, 431.4), 248.1 U/L (237.5, 266.6), respectively; *P* < 0.001).

CK-18 fragments levels ranged from 188.5 to 563.4 U/L (median (Q25, Q75): 294.5 U/L (243.8, 388.5)) and the levels were significantly higher in patients with NASH than that in the non-NASH group (median (Q25, Q75): 372.9 U/L (319.6, 431.4), 248.1 U/L (237.5, 266.6), respectively; *P* < 0.001) (Figure 1). 

The CK-18 fragment levels showed a significant positive correlation with steatosis severity (r = 0.492, *P* <0.001), ballooning (r = 0.211, *P* < 0.041), lobular inflammation (r = 0.346, *P* = 0.001), and fibrosis stage (r = 0.407, *P* < 0.001).

### Multivariate Analysis

Multivariate analysis includes a model that combines BMI, WHR, AST, ALT, ALB, ALP, γ-GT, platelets, UA, hs-CRP, TG, and CK-18 fragments. ALT [OR = 1.078, (95% CI: 1.020 - 1.139), *P* = 0.007], platelets [OR = 1.013, (95% CI: 1.001 - 1.025), *P* = 0.040], CK-18 fragments [OR = 1.012, (95% CI: 1.003 - 1.021), *P* = 0.011], and TG [OR = 1.006, (95% CI: 1.001 - 1.012), *P* = 0.019] were independent variables in patients with NASH (Table 3). The AUROC curves were 0.811 (95% CI: 0.722 - 0.899), 0.631 (95% CI: 0.515 - 0.746), 0.892 (95% CI: 0.824 - 0.960) and 0.714 (95% CI: 0.611 - 0.818) for the ALT, platelets, CK-18 fragments, and TG for the prediction of NASH (Table 3).

CK-18 fragment levels showed a significant positive correlation with ALT (r = 0.639, *P* < 0.001) and TG (r = 0.390, *P* < 0.001). No correlation was found with platelets (*P* = 0.645).

**Table 3:** Multivariate analysis according to the severity of NASH patients.

**Factor**	***P* value**	**OR**	**OR 95% CI**	**AUROC**	**AUROC 95% CI**
ALT(U/L)	0.007	1.078	1.020 - 1.139	0.811	0.722 - 0.899
Platelets(x 10^9^/L)	0.040	1.013	1.001 - 1.025	0.631	0.515 - 0.746
CK-18(U/L)	0.011	1.012	1.003 - 1.021	0.892	0.824 - 0.960
TG(mg/dL)	0.019	1.006	1.001 - 1.012	0.714	0.611 - 0.818

Abbreviations: ALT, alanine aminotransferase; CK18, Cytokeratin 18; TG, triglycerides;

### Model to predict NASH in NAFLD patients

**Figure 2:**
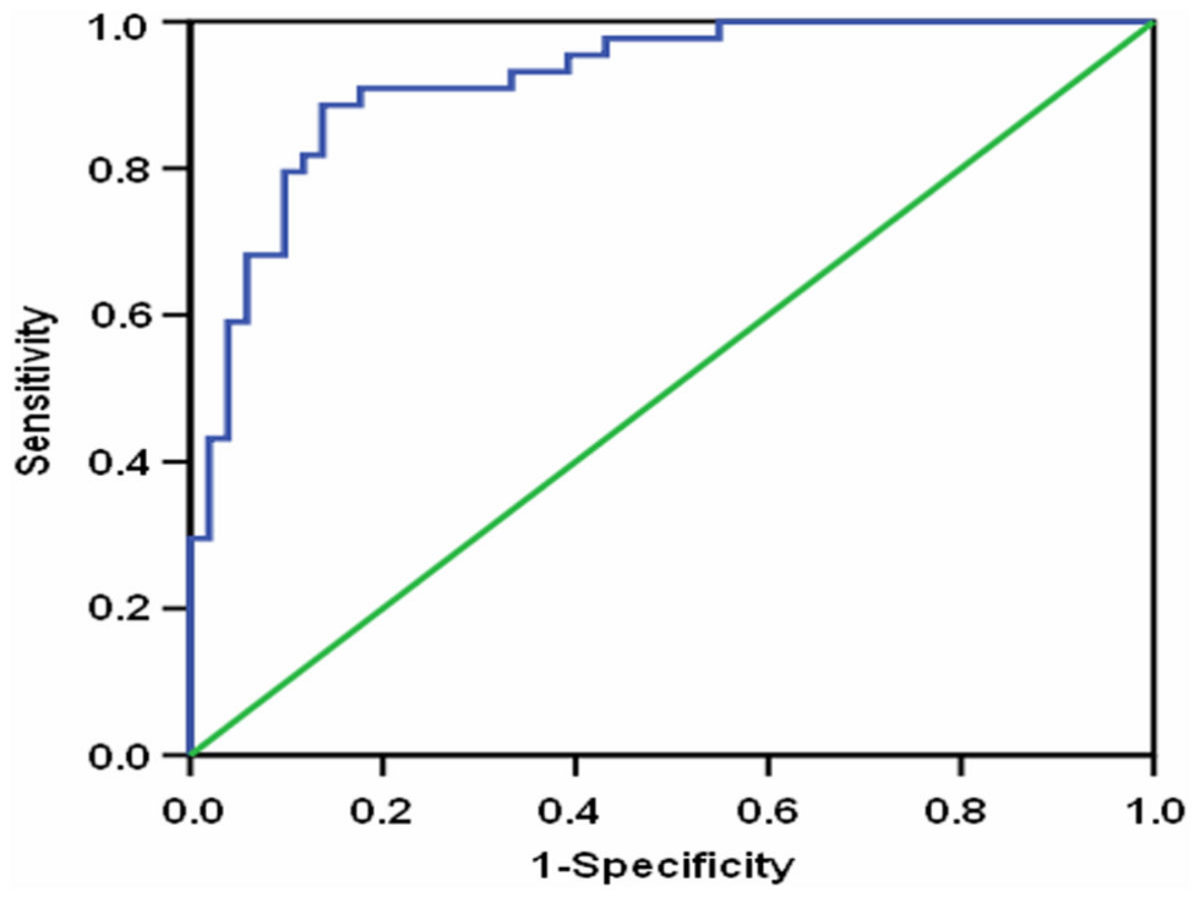
The model receiver operating characteristic (ROC) curve for the definitive NASH diagnosis in NAFLD patients.

A new model that combines ALT, platelets, CK-18 fragments, and TG was established through logistic regression in the NAFLD patients. The equation of this model was: -12.764 + 0.075 x ALT (IU/L) + 0.013 x platelets (×10^9^/L) + 0.012 x CK-18 fragment levels (U/L) + 0.006 x TG (mg/dL). The AUROC curve for the prediction of NASH was 0.920 (CI 0.866 - 0.974) (Figure 2). A cutoff value of 0.361, with a sensitivity of 89% and a specificity of 86%, has a positive predictive value of 89% and a negative predictive value of 89%. 

## Discussion

 Long-term longitudinal studies suggest that NASH patients might have higher mortality and are more likely to acquire liver-related complications compared with patients with simple steatosis, which was considered benign and non-progressive. Recent studies demonstrated that patients with simple steatosis might develop NASH [32]. Therefore, a prompt and accurate diagnosis of NASH and regular follow-up are considered significant in predicting the development of NAFLD.

Analyses of the relationships between clinical biomarkers and NAFLD were performed in a number of studies. Kashyap et al. reported that the TG, AST, and ALT levels increased significantly with the progression of NAFLD [31]. Raza et al. observed that the prevalence of DM differed among NASH and simple steatosis patients [33]. Feldstein et al. reported that AST levels were significantly higher in subjects with NASH than those without NASH [25]. Liu et al. demonstrated that hemoglobin is expressed by hepatocytes, whereas oxidative stress upregulates its expression in NASH [19]. Sirota et al. confirmed that an elevated UA level was associated with increasing severity of NAFLD on ultrasonography [34]. Iron overload was significantly associated with NAFLD in obese patients as represented by ferritin elevation, which might damage hepatocytes [20]. Serum ferritin was an independent predictor of histologic severity and advanced fibrosis in patients with NAFLD [35]. Decrease in platelet count was reported as a risk factor for advanced fibrosis [3]. Age, BMI, cholesterol, glucose, insulin, glycohemoglobin, and homeostasis model assessment of insulin resistance values significantly increased in NASH patients [36,37]. These studies were conducted in European countries with different grouping criteria. The routine laboratory examination indexes were not consistently identical with the staging of NAFLD. Various differences between Chinese and European, such as diet habit, regional difference, racial and ethnic difference, would present additional differences. Furthermore, NAFLD was usually diagnosed through physical examination in China. Therefore, we selected the relevant markers such as age, gender, smoking habits (general character), BMI, WHR (obesity), SBP, DBP (hypertension), TG, TC (lipid metabolism), FG (glucose metabolism), hs-CRP (inflammation), AST, ALT, ALB, bilirubin, ALP, γ-GT, INR (liver reserve function), hemoglobin (oxidative stress), platelets, WBC, creatinine, UA, and ferritin (risk factors). Compared with non-NASH patients, NASH patients were heavier with more severe liver injury and higher levels of UA, hs-CRP and TG, which were easily affected by race, age, gender, and diet. This study found that the platelets of NASH patients were higher than that of non-NASH patients. However, some studies reported a decrease in platelet count in patients with NASH or severe fibrosis [38-40]. Fitzpatrick et al. found no statistical difference on platelets of non-NASH and NASH patients [41]. Such findings may be associated with small sample size and single-center study.

CK-18 is crucial in the mechanism of chronic liver disease, especially in NAFLD [27]. The levels of CK-18 fragments in this study were determined by ELISA. Results show that NASH patients have higher levels of CK-18 fragments, which were closely correlated with steatosis severity, ballooning, lobular inflammation, and fibrosis stage in the NAFLD patients. Results indicate that the serum CK-18 fragments could serve as a biomarker to predict the severity of NAFLD.

We selected multiple serologic markers to identify NASH from NAFLD because specific markers in diagnosing NASH are lacking. Several noninvasive tests were conducted to predict or diagnose NASH, such as the Nice model [42]. Palekar et al. established a composite index to distinguish simple steatosis from NASH, which was calculated by summing the risk factors [43]. Hypertension, diabetes, AST>27 IU/L, ALT>27 IU/L, obstructive sleep apnea, and non-black race were used to establish a clinical scoring system of NASH by Campos et al. [38]. Gholam et al. found that the combination of liver injury markers (AST, ALT) and hyperglycemia markers (glycosylated hemoglobin, presence of diabetes) could accurately predict the presence of NASH and fibrosis [44]. Poynard et al. developed and validated NASH test as a new biomarker of NASH in patients with NAFLD [45]. Francque et al. found a new formula, which was composed of the levels of ALT, fasting levels of C-peptide, and ultrasound steatosis scores for predicting NASH [46]. The research respondents listed above were mostly inpatients in non Asia-countries. Most of them have morbid obesity. A recent study reported that the circulating levels of two apoptotic markers, namely, CK-18 fragments and soluble Fas, accurately predict the presence of NASH [47]. However, this study suggests that apoptotic markers alone could not sufficiently predict NASH because apoptosis is only one of the complicated mechanisms of NASH. Wong et al. demonstrated a two-step approach to improve the accuracy of diagnosing NASH using CK-18 and fibroblast growth factor 21, which reflected different aspects of the pathogenesis of NAFLD [48]. However, the approach was difficult in practice because of the limitation on clinical detection. Biomarkers that are simple, feasible, and inexpensive, are more widely accepted in China. Some markers in previous noninvasive scoring systems were not tested frequently or only detected in the scientific researches. The methods concerned a tiny fraction of the pathogenesis of NAFLD. Therefore, these non-invasive scoring systems are not suitable for Chinese. 

This study established a new scoring system, which includes ALT, platelets, CK-18 fragments, and TG to diagnose NASH in NAFLD patients. The diagnostic accuracy of this model was better than the use of CK-18 fragments, ALT, platelets, or TG alone. The cutoff value of 0.361 had a sensitivity of 89%, a specificity of 86%, a positive predictive value of 89%, and a negative predictive value of 89%. Therefore, this model could be regarded as a novel noninvasive test to substitute for liver biopsy in identifying NASH. 

The limitations of the study, such as the relatively small number of non-NASH patients, single-center study with single race, and the lack of validation in an independent cohort, could affect the statistical power of analyses. Therefore, a large-scale, multi-center, multi-ethnic, and validation cohort study is necessary for future NAFLD studies.

In conclusion, NASH subjects showed higher serum CK-18 fragments. The levels were positively correlated with liver histologic manifestations such as steatosis, ballooning, lobular inflammation, and fibrosis. A noninvasive scoring system including ALT, platelets, CK-18 fragments, and TG predicted NASH in NAFLD patients in China. 
